# Delayed Surgical Treatment in Patients with Chronic Carpal Tunnel Syndrome Is Still Effective in the Improvement of Hand Function

**DOI:** 10.3390/medicina59081404

**Published:** 2023-07-31

**Authors:** Marta Twardowska, Piotr Czarnecki, Marta Jokiel, Ewa Bręborowicz, Juliusz Huber, Leszek Romanowski

**Affiliations:** 1 Department of Traumatology, Orthopaedics and Hand Surgery, Poznan University of Medical Sciences, 28 Czerwca 1956 r. Street, No. 135/147, 61-545 Poznan, Poland; piotr_czarnecki@tlen.pl (P.C.); marta.jokiel@gmail.com (M.J.); ewabreborowicz@gmail.com (E.B.); lromanowski@orsk.pl (L.R.); 2 Department of Pathophysiology of Locomotor Organs, Poznan University of Medical Sciences, 28 Czerwca 1956 r. Street, No. 135/147, 61-545 Poznan, Poland; juliusz.huber@ump.edu.pl

**Keywords:** chronic carpal tunnel syndrome, median nerve neuropathy, delayed surgery, effectiveness of treatment, functional tests, questionnaires evaluation

## Abstract

*Background and Objectives*: Severe carpal tunnel syndrome (CTS) is the most common compression neuropathy in the upper extremities treated conservatively; later, when advanced, CTS is treated mostly surgically. The most prevalent symptoms comprise numbness, as well as sensation loss in the thumb, index, and middle finger, and thenar muscle strength loss, resulting in impaired daily functioning for patients. Data on the results of CTS treatment in patients with delayed surgical intervention are scarce. The aim of this study was to determine the postoperative results of chronic carpal tunnel syndrome treatment in patients with symptoms lasting for at least 5 years. *Materials and Methods*: A total of 86 patients (69 females, 17 males) with a mean age of 58 years reporting symptoms of CTS for at least 5 years (mean: 8.5 years) were prospectively studied. The average follow-up time was 33 months. All patients underwent the surgical open decompression of the median nerve at the wrist. A preoperative observation was composed of an interview and a clinical examination. The subjects completed the DASH (the Disabilities of the Arm, Shoulder, and Hand), PRWE (Patient-Rated Wrist Evaluation), and self-report questionnaires. Global grip strength, sensory discrimination, characteristic symptoms of CTS, and thenar muscle atrophy were examined. Postoperatively, clinical and functional examinations were repeated, and patients expressed their opinions by completing a BCTQ (Boston Carpal Tunnel Syndrome Questionnaire). *Results*: We found improvements in daily activities and hand function postoperatively. Overall, 88% of patients were satisfied with the outcome of surgery. DASH scores decreased after surgery from 44.82 to 14.12 at *p* < 0.001. PRWE questionnaire scores decreased from 53.34 to 15.19 at *p* < 0.001. The mean score of the BCTQ on the scale regarding the severity of symptoms was 1.48 and 1.62 on the scale regarding function after surgery. No significant differences were found in the scores between the male and female groups or between age groups (*p* > 0.05). A significant increase in global grip strength from 16.61 kg to 21.91 kg was observed postoperatively at *p* < 0.001. No significant difference was detected in the measurement of sensory discrimination (6.02 vs. 5.44). In most of the examined patients, night numbness and wrist pain subsided after surgery at *p* < 0.001. Thenar muscle atrophy diminished after surgery at *p* < 0.001. *Conclusions*: Most patients were satisfied with the results of CTS surgery regarding the open decompression of the median nerve even after 5 years of ineffective conservative treatment. Significant improvement of the hand function was confirmed in the functional studies.

## 1. Introduction

Carpal tunnel syndrome (CTS) is the most common compression neuropathy in the upper extremity, affecting 2–4% of the population [1]. Compression of the nerve causes numbness in the middle finger, index finger, and thumb, diminished sensation, and atrophy of the thenar muscle, which leads to deterioration in the patient’s daily living activities [2]. Finger numbness often occurs at night, causing difficulties in sleeping. Risk factors for CTS may include rheumatoid diseases, diabetes, bone fractures, using a computer, obesity, hypothyroidism, and hormone replacement therapy [2,3,4]. However, in most cases, the cause is unexplained, leading to idiopathic CTS [5].

A detailed patient history and clinical examination are required for CTS diagnosis. Sometimes, they are sufficient for a correct diagnosis confirming CTS [5,6]; additional methods may include an X-ray, ultrasound, and nerve conduction studies (electroneurography or electromyography) [7,8,9,10]. These tests can help rule out other causes of patient complaints.

Treatment for CTS may be non-operative initially, involving physiotherapy (e.g., manual therapy and neuromobilization), the use of orthoses, or corticosteroid injections, although surgical treatment is indicated for patients with advanced disease severity [5]. Possible surgical techniques are open surgery or endoscopic techniques [6]. Both methods involve transection of the flexor retinaculum, thus decompressing the median nerve [11]. There are randomized controlled trials and numerous observational studies in the literature which have confirmed the differences and similarities in postoperative results between open and endoscopic methods of median nerve decompression yield [11,12].

There are scarce data on the results of CTS treatment in patients with delayed surgical intervention. The rationale of this study was to provide data that could help to discuss the prognosis and results of treatment in these patients. The aim of this study was to determine the postoperative clinical and functional results of surgery in chronic carpal tunnel syndrome treatment cases, i.e., those patients for whom the above symptoms have lasted for at least 5 years, which is a novelty in the literature on the topic. We used a set of both clinical and questionnaire tools which have never been reported before. The null hypothesis is that CTS treatment in patients with delayed surgical intervention does not bring the significant improvement. The secondary aim of the study was to age-wise and gender-wise evaluate the results with the hypothesis that age and gender do not influence the results of the treatment.

## 2. Materials and Methods

### 2.1. Subjects, Study Design, and Clinical Evaluation

This study was performed on 114 patients diagnosed with chronic carpal tunnel syndrome (CCTS) who underwent surgery in the Traumatology, Orthopedics, and Hand Surgery Department Poznan University of Medical Sciences, Poland, from 2014 to 2018. The study was approved by the Bioethics Committee at the Poznan University of Medical Sciences (Resolution no. 32/15).

The diagnosis of CTS was confirmed by a medical team (with at least 5 experienced hand surgeons) during clinical meetings. Patient history was analyzed, with patients exhibiting characteristic symptoms previously described in the literature. The Phalen test and Tinnel–Hoffman sign had to be positive. We considered these two clinical tests reliable enough to confirm the symptoms of the carpal tunnel syndrome and did not perform any other clinical tests like the reversed Phalen or Durkan’s test. The combination of the Tinel–Hoffmann sign together with Phalen test proved to be of high sensitivity and specificity—similar to the Phalen and Durkan test combination [13,14].

We considered the minimum duration of symptoms before surgery for 5 years because such a period causes permanent damage to the nerve, so the benefits of surgical treatment can potentially be less satisfactory than in patients treated earlier.

The study group consisted of patients who, despite the use of various conservative methods (involving physiotherapy, e.g., manual therapy and neuromobilization, the use of orthoses, or corticosteroid injection), still reported severe symptoms of CTS and therefore qualified for surgical treatment. During the study, electrophysiological test results were not provided because this was not the purpose of the study. In our department, imaging studies and nerve conduction studies are not obligatory for carpal tunnel diagnosis. These modalities are used in revision cases or in multiple crush syndromes [15,16], which were excluded from this study.

After the diagnosis was verified, patients qualified for open median nerve decompression surgery. All patients underwent the surgery in the same hospital according to the department’s approved procedure. Subsequently, all patients underwent a follow-up examination in 2017–2019, which was conducted a minimum of 12 months after surgery. At the end of 2019, some of the patients might have already been the first victims infected with COVID-19, which is known for causing polyneuropathies; therefore, we accepted this year as the ending point of the follow-up [17,18,19].

**Figure 1:**
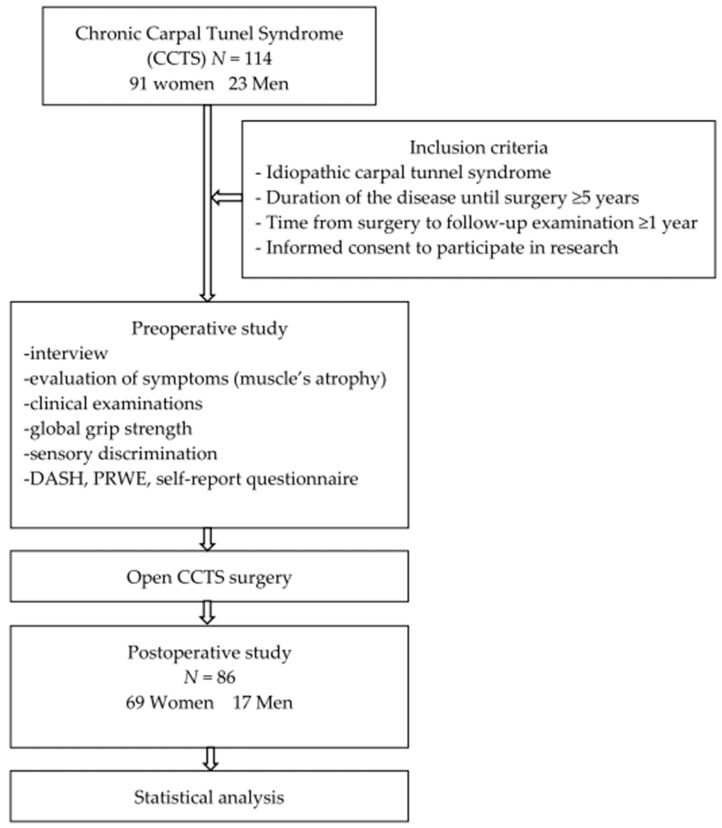
Flow chart detailing the research procedure.

The preoperative examination included a group of 114 patients, but some of them did not meet the inclusion or exclusion criteria or did not attend the follow-up examination after surgery because of COVID-related problems (Figure 1). Finally, 86 patients qualified for the second stage of the study (Table 1). In cases of bilateral CTS, only one hand with more severe symptoms was operated on and evaluated in the period of follow-up.

However, the long time between the decision for surgical treatment and the performance of surgery was not due to health system organizational reasons. The most common reasons for delaying the surgical CTS procedure reported by the patients were fear of the procedure, waiting for improvement from conservative treatment, and the lack of a precise diagnosis of the disease.

This study was prospective and included two stages. The first involved a preoperative examination. Patients underwent a clinical examination, and global grip strength and sensory abnormalities were evaluated.

The inclusion criteria were as follows:

- Idiopathic carpal tunnel syndrome;
- Duration of the disease until surgery ≥5 years;
- Time from surgery to follow-up examination ≥1 year;
- Informed consent to participate in research.

The exclusion criteria were as follows:

- Diagnosed diseases of the cervical spine, e.g., consequences of mechanical injuries, disc-root conflicts, or degenerative disease of the spine during treatment;
- Previous injuries within the examined upper extremities and diseases that may affect the function of the median nerve, e.g., bone fractures, consequences of upper limb ischemia, thoracic outlet syndrome, and shoulder injuries;
- Patients with previous carpal tunnel syndrome surgeries or multiple injections of corticosteroids,
- Symptoms of other concomitant hand problems like triggering of the fingers, thumb carpometacarpal (CMC) I joint arthritis;
- The presence of polyneuropathy symptoms.

On the basis of medical data obtained from hospital records and the medical history collected during the study, we created a database containing information on the characteristics of symptoms, conservative treatment, type of work, comorbidities, and other relevant information. During the clinical examination, we examined the Phalen and Tinnel–Hoffman signs, and we assessed the thenar muscle mass. The thenar muscle mass was comparatively visually inspected before and after the surgery on a two-stage scale: 1—normal and 0—thenar muscle atrophy. The global grip strength test consisted of measuring force with an electronic dynamometer, i.e., the Biometric LTD Hand Kit. The patient made three attempts to tighten the handle of the dynamometer with the highest possible force, after which the average of the three results was calculated. The test took place in a standing position, with the elbow joint placed against the torso and bent at an angle of 90°; then, the test hand tightened the dynamometer handle. The wrist had to be positioned at 0° or in slight dorsiflexion (approximately 10–20°) to offset the effect of the flexor retinaculum on grip strength. Sensation was measured using a two-point discrimination (2PD) test [20]. It was carried out using the so-called Weber’s compass, which allows the fingertips of the examined patient to be touched in two places at the same time. The shortest distance between two points that can be recognized by the patient as two separate points is the measure of discrimination. Under normal conditions, it ranges from 3 to 6 mm. Results above 15 mm refer to an undeterminable resolution of sensation.

### 2.2. Questionnaires and Surgery

Patients completed the DASH (the Disabilities of the Arm, Shoulder, and Hand) and PRWE (Patient-Rated Wrist Evaluation) questionnaires before the operation. After the operation, in addition to these two questionnaires, they also completed the BTCQ (Boston Carpal Tunnel Syndrome Questionnaire). All questionnaires have been validated in and adapted to Polish [21,22].

The DASH questionnaire consists of 30 questions, of which 21 concern the assessment of limb function, and 6 questions concern symptoms such as pain, tingling, weakness, and stiffness [23]. The next 3 concern interpersonal relationships and self-perception. The questionnaire ranges from a scale of 1–5. To be able to calculate the result of the questionnaire, the respondent must answer at least 27 out of 30 questions. The responses are summed up, and the resulting sum is divided by the number of answers given, obtaining an average answer in the range from 1 to 5. The obtained result is transformed to a 100-point scale by subtracting 1 and multiplying by 25. The above transformation is intended to facilitate comparisons with other scoring questionnaires in the range from 0 to 100. All questions are about complaints in the last week.

The PRWE questionnaire assesses the level of pain and the degree of difficulty caused by specific activities to the patient [24].

It consists of 15 questions. It is divided into 2 parts:

- The first consists of 5 questions about the intensity of hand pain.
- The second consists of 10 hand function assessment questions.

In both parts, the patient evaluates the average severity of symptoms over the last week. The patient marks the average of symptoms on a scale from 0 to 10, where 0 means no pain or no difficulty in performing the given activities, and 10 means the greatest pain experienced or always present pain or impossible activity.

After the examination, patients underwent open decompression surgery, which involved surgical incision of the flexor retinaculum and releasing the median nerve compression. The duration of the surgery, including anesthesia, was 45 min on average. We used a tourniquet during surgery for 30 min, but it was deflated before the closure to check hemostasis. The surgery was performed with the brachial plexus anesthesia routinely utilized in out department during CTS surgery. A longitudinal incision approximately 2–3 cm long was made on the palmar surface above the carpal tunnel, distally to the wrist flexion crease. After skin incision, the palmar aponeurosis was cut, followed by a flexor retinaculum cut in the proximal and distal directions. A complete cut was verified with scissors. Hemostasis was then performed, and a skin suture was placed using absorbable thread (Rapid 3/0 or 4/0-Yavo Poland). All patients underwent the same skin closure method. We did not apply plaster immobilization, only a soft dressing.

The patients were followed up for a minimum of one year. Out of 114 patients, 28 did not participate in follow-up for various individual reasons (e.g., refusal to come for examination, death, other medical conditions). In addition, some data were missing in pre-op and post-op documentation (grip strength and 2PD examination). The second stage of the study included a post-operative follow-up examination of the operated hand, during which, in addition to the above, patients completed the BCTQ. The BCTQ is a dedicated survey for CTS patients [23,25,26]. The questionnaire consists of two parts. The first of them, called the Symptom Severity Scale (SSS), contains 11 questions concerning the frequency and perception of pain by the patient day and night, numbness and tingling of the fingers, as well as difficulties in gripping small objects such as a pen or keys. The second part, called the Functional Status Scale (FSS), consists of 8 questions about the patient’s functioning and the degree of difficulty with everyday activities. In each part, the patient selects the answers on a scale of 1–5, where 1 means the least severity of symptoms or no difficulties with a given activity, and 5 means the most severe symptoms or inability to perform activities.

The patients were additionally asked about satisfaction with the result of the operation (yes, no, hard to say), and then there were open-ended questions where the patients could describe their symptoms and whether they changed over time. We also asked if the patient was still working and after what time he or she returned to work.

### 2.3. Statistical Analysis

On the basis of medical data obtained from hospital records and our own measurements, a database was created. The data were then statistically analyzed using Statistica 13.0; TIBCO Software Inc., Palo Alto, CA, USA (2017). Statistically significant results were considered those in which the significance level (*p*) was below 0.05.

Tests were selected according to the distribution of the variables. The absence of a normal distribution in the study group was established using the Shapiro–Wilk test.

According to the distribution of parametric and nonparametric tests, the following tests were used:

- Parametric tests, when the variables met the assumptions of a normal distribution: Student’s *t*-test for dependent variables, and Student’s *t*-test for the independent variables;
- Non-parametric tests when variables did not meet the assumptions of normal distribution: Wilcoxon test for dependent variables, Mann–Whitney U test for independent variables.

When comparing more than two groups at the same time, Kruskal–Wallis nonparametric tests were used in addition to Dunn’s post-hoc tests. When analyzing related variables on a nominal scale (in the case of Tinel’s and Phalen’s characteristic symptoms), the chi-squared test was used.

When the distribution was normal, the mean and standard deviation were used; when the data did not follow a normal distribution, the median and quartile range were used.

For grip strength, the 2PD Wilcoxon Matched Pairs Test was used. For the PRWE, DASH, BCTQ, and age-wise and gender-wise comparisons, the Mann–Whitney U Test was used. Spearman’s rank correlation was used for correlations calculations between the duration of symptoms, follow-up, and PRWE, DASH, and BCTQ questionnaires. Kruskal–Wallis tests were used for the analysis of self-reported questionnaire answers in terms of symptom duration.

## 3. Results

The dominant hand in 93% of patients was the right hand. In 47% of patients, the left hand was operated on; 53% had their right hand operated on. No trigger fingers were reported or detected postoperatively by the patients.

Most of the patients were satisfied with the outcome of the surgery. In total, 74 people (88% of the surveyed subjects) reported that they were satisfied with the result of the surgery, with only five patients reporting that the result of the procedure was not satisfactory.

Before the surgery, 39 subjects (49%) exhibited atrophy of the thenar muscles, and after the operation, atrophy was found in 29 subjects (36%), i.e., a significant improvement (regeneration of the muscle mass) was observed in 13% of the subjects (*p* < 0.001). The time when thenar muscles were regenerated postoperatively was about 24 months on average.

A significant (*p* < 0.001) increase in global grip strength from 16.61 ± 8.47 kg to 21.91 ± 8.26 kg was also noted (Table 2). No significant difference was observed in the measurement of sensory discrimination. Before surgery, patients revealed scores of 6.02 ± 2.51 mm, whereas after surgery, they had scores of 5.44 ± 1.66 (Table 3).

Patients with chronic CTS demonstrated significant improvements in performing daily activities and hand function postoperatively (Table 4, Table 5 and Table 6).

Significantly (*p* < 0.001) lower DASH questionnaire scores were observed in patients after surgery compared with patients before surgery. DASH scores decreased from 44.82 ± 18.14 to 14.12 ± 18.10 (Table 4). PRWE questionnaire scores significantly (*p* < 0.001) decreased from 53.34 ± 21.17 to 15.19 ± 22.45 (Table 5).

The mean score of the BCTQ for all subjects on the scale regarding the severity of symptoms was 1.48 ± 0.75 after surgery, and on the scale regarding function, it was 1.62 ± 0.85. There were no statistically significant differences in the scores between the male and female groups or between age groups (*p* > 0.05) (Table 6).

Chronic CTS patients’ surgical results indicated that there was no correlation between the duration of symptoms and the questionnaire results (Table 7).

The correlation between the score of the questionnaires and the number of months between surgery and follow-up was also checked. Post-operative DASH, PRWE, and BCTQ scores improved with the number of months to wait for a follow-up visit.

Analysis of the questions about satisfaction after surgery in terms of symptom duration found that there was no significant interaction effect between these factors.

The time after which symptoms were retreated and patients’ satisfaction was raised was 12 months on average.

## 4. Discussion

This study provided evidence on the positive results of CTS treatment in patients with delayed surgical intervention. Regarding improvements in daily activities and hand function postoperatively, we can conclude that 88% of patients were satisfied with the surgical outcomes. DASH scores decreased and PRWE questionnaire scores decreased significantly at *p* < 0.001. The same holds true for the BCTQ score regarding the severity of symptoms and improvement of the hand function after surgery. Functional studies revealed an increase in global grip strength postoperatively at *p* < 0.001, while no significant difference was detected in the measurements of sensory discrimination.

The purpose of this study was to answer the question of what results could be expected from delayed surgery in patients with chronic carpal tunnel syndrome, and what patients could expect from such surgery. The group consisted of people who had the condition for a minimum of 5 years (in a previous study, it was an average of 9 years). Many of these people had been treated conservatively, but despite the use of various methods (e.g., wrist orthoses, manual therapy, and injections), the complaints were unable to be resolved; thus, this study did not analyze those methods [27]. The average age of the patients was 58; therefore, the statistical analysis divided the group into those under and over 60. This age is also often the limit of professional activity, which may reflected the results in these two age groups.

Although the endoscopic method of carpal tunnel decompression has become increasingly popular in recent years, the open method is still just as effective. Publications from 2004 to 2020 have indicated that the endoscopic method reduces hospitalization time and pain around the scar [11,12,28]. Long Chen’s study of 1596 patients indicated that both methods showed similar results in the resolution of symptoms, but patients treated with the endoscopic method regained better function in the hand faster and returned to work sooner [29].

There are several answers to the question of what kind of surgical results we can expect in patients with short surgical waiting times in the literature, which can be referred to when analyzing selected parameters. One such paper was published by Louie et al., which reported a study conducted on 211 patients with a minimum follow-up period of 10 years (from 11 to 17 years), confirming the prior finding that most patients are satisfied after open median nerve decompression, even in the long term after surgery [30].

Considering the data presented in Table 7, it can be concluded that it takes a long time for hand function to improve, and one year after surgery may sometimes be too short of a period to regain satisfactory improvement. However, it should be noted that although the correlation results were statistically significant, the correlation coefficients were very low; therefore, the above conclusions may not be sufficiently significant.

### 4.1. Patient’s Satisfaction

One of the main goals of this study was to answer the question of whether patients would be satisfied after surgery, and whether the outcome of surgery would be satisfactory to them. Patients were mostly satisfied with the outcome of the surgery after receiving treatment for CCTS. They mentioned the elimination of night-time numbness and improved sensation and strength in the hand as the main reasons for satisfaction, among others. Those who were dissatisfied with the surgery also sometimes felt that there was an improvement in overall hand function or the elimination of night-time numbness. Patients cited a thick scar or tingling around the scar as reasons for dissatisfaction. In addition, in the literature, most patients were satisfied with the outcome of surgery, such as in the study by Louie mentioned above [30]. In the study by Michelotti et al., a numerical scale from 1 to 100 was used to assess patient satisfaction (the average score was 90.33). Such an assessment seems more detailed, but for a patient, the answer to a yes or no question may be more obvious. [31] Among the other issues addressed in the Japanese study were factors affecting satisfaction with surgery [32]. In the study, the authors showed that age and depression were factors that significantly affected satisfaction with the outcome of surgery. Other studies have also highlighted the significant impact of psychological factors on satisfaction, such as the work by Maempel, who studied 809 patients, of which 674 said they were satisfied with the surgery [32]. In his paper, he reported that although people in a worse mental state had lower levels of satisfaction, most such people were generally satisfied with the outcome of surgery.

### 4.2. Thenar Muscle Atrophy

Another parameter measured was the evaluation of the thenar muscles. One of the main muscles is the short thumb abductor muscle (Latin: Musculus abductor pollicis brevis: APB). It is located most superficially; therefore, in cases of prolonged compression of the median nerve, the APB may undergo atrophy, which, in the clinical picture, will result in emaciation of the thenar muscles. In our study, such atrophy was observed in 49% of patients with CCTS before surgery. However, studies on CCTS have clearly shown that atrophy of the APB muscle in particular is very common [33,34]. Moreover, because of this, many patients lose the ability to oppose their thumb, an activity which is essential for daily functioning. In one study, APB atrophy after surgery was found in 36% of the subjects. In such cases, the literature suggests performing surgery to regain thumb opposition, such as tendon transfer of the palmaris longus muscle (PL) using the Camitz technique [33,35] or modified Camitz technique [36]. In our study, we used a self-reported scale to assess thenar muscles atrophy, where 1 meant normal thenar muscles and 0 meant thenar muscle significantly denervated.

### 4.3. Hand Grip Strength

Another characteristic symptom reported by patients with CTS is weakened strength in the hand, which often results, for example, in patients dropping objects [37]. One reason for this weakness is leanness of the APB. In this study, a dynamometer was used to measure global grip strength. Weakened grip strength can affect up to 71% of people with CTS (according to a study on 172 patients) [38]. One limitation of this measurement is the large effect of flexor muscles on global grip strength.

In the present study, a significant improvement in grip strength was observed in all of the studied patients. This improvement was statistically significant in women; however, no such improvement was observed in the male group. Studies on the electrophysiological measurements of the APB have also indicated improved parameters after surgery [34]. Capasso’s work on severe CTS demonstrated that the strength of the hand improved after surgery [34]. With a significant improvement in strength in the hand, post-operative patients can perform more activities not possible before surgery, which certainly affects their satisfaction with the operation. Lai and his team tested whether flexor retinaculum reconstruction can affect grip strength and hand function (measured using the BCTQ) [39]. After analyzing 615 patients, they noted that such modification had no effect on global grip strength or SSS scores relating to symptom severity. The only significant findings were improvements in scores on the FSS related to patient functioning. In a prospective study by Michelotti et al. on outcomes after open CTS surgery, no statistically significant improvements were seen in measures of strength [40]. In the study group of 30 subjects, the average strength before surgery was 4.52, whereas 2 years after surgery, it was 4.9.

### 4.4. Sensation Studies Results

Sensory two-point discrimination (2PD) was measured using a Weber discriminator; the values are given in millimeters. In some subjects, the result was >15 mm (indeterminate sensory resolution). In this group of subjects, 15 mm was taken as the result for the purpose of statistical calculations.

In this study, in the measurement of the mean discrimination on the fingers of the operated hand, no significant improvement was observed; sensation on the fingertips remained comparable to that before surgery. Several results in the literature also showed no statistically significant improvement in 2PD scores. A study by Bai et al. evaluating 85 patients before and after CTS surgery (follow-up: 12 months after surgery) reported the following results: 6.9 mm before surgery and 3.1 mm after surgery [41]. A study by Wessel et al. involving 73 patients examined before and after surgery, in follow-up examinations performed, on average, more than 12 months after surgery, also showed no significant improvement in the 2PD test [20]. Before surgery, the average was 7.4 mm, whereas after surgery, it was 6.4 mm.

Although sensory abnormalities on the fingers affect the vast majority of patients with CTS (70–87%), the above studies indicate that sensory resolution is not an indicator of the surgical outcome [38]. Although some patients showed an indeterminate resolution, they were able to define a single-point sensing, which we interpreted as protective sensation, which is often sufficient for daily functioning. Discrimination sensation, however, is less important than the protective sensation necessary for daily functioning.

### 4.5. Specific Symptoms

During the physical examination in medical practice, the Tinel’s sign and Phalen’s sign, due to their ease of performance, are among the tests most often performed during the diagnosis of CTS. These tests are not highly sensitive and specific; therefore, a positive result does not always mean a diagnosis of CTS.

In patients with CTS, the Tinel’s sign has a lower sensitivity and specificity than the Phalen’s sign. Hegmann’s study, mentioned above, showed a sensitivity of Phalen’s sign of 52.8% and Tinel’s sign of 37.7% [42]. A tingling sensation in the hand waking patients from sleep was reported by 77.4% of people. In contrast, George Phalen’s original study of 654 patients showed the sensitivity of Phalen’s symptom to be 74% [43].

In this study, the majority of patients were positive for both tests before surgery, which indeed became negative after surgery.

### 4.6. Questionnaires

To assess hand function, patients completed three questionnaires. A decrease in the questionnaire score after surgery indicated an improvement in overall function. In the literature, the Boston Carpal Tunnel Questionnaire is most commonly used to assess the severity of symptoms and hand function [44]. The other tests are also suitable for reliably assessing patients with CTS, but the BCTQ is the shortest of them. Greenslade, in his study, compared the time it took patients to complete questionnaires assessing hand function [27]. The DASH questionnaire took an average of 6.8 min, whereas the BCTQ took 5.6 min. In this study, nonparametric tests were used to calculate the final results of the questionnaires; the medians and quartile ranges are reported in the tables. However, the results of the mean are discussed further because these values are most commonly reported in the literature. In this study, the mean score of the DASH questionnaire decreased from a preoperative score of 44.82 to 14.12 after surgery, which means that patients reported fewer symptoms after surgery and their upper limb function improved. Patients under 60 years of age had better results, meaning they regained better function than the older group of patients. The results of the questionnaire from the work and sports/playing an instrument module were not included in this paper due to the insufficient number of completed questionnaires. In the publication by Bai et al. [41] regarding 85 patients with CTS lasting approximately 6 months, the DASH score before surgery was 35.1, whereas after open median nerve decompression surgery, it was 10.4; thus, each group had better scores, i.e., greater dexterity in the hand, than the patients in our study. In the BCTQ, they also had better scores: before the operation, the mean score on the SSS was 2.7, and on the FSS, it was 2.4; after the operation, the mean score on the SSS was 1.3, and on the FSS, it was 1.1, although the improvement in scores on this questionnaire was not statistically significant.

In the results of the PRWE questionnaire, the whole group significantly improved after surgery, i.e., their wrist function increased. In addition, this questionnaire showed that after surgery, statistically better results were recorded in the group of patients under the age of 60 than in the older group. The only publication on patients with CTS who completed the PRWE questionnaire concerns conservative treatment [45]. In the literature, this questionnaire is mainly used to assess wrist function after fractures of the radius and elbow; thus, after reviewing the publications, its selection for the evaluation of patients with CTS may not be appropriate [46].

The BCTQ is a questionnaire that is dedicated to patients with CTS.

Our study was conducted in a group of patients after surgery. In Louie’s study, the mean of the BCTQ (published as the Levine-Katz scale) was 1.3 on the SSS (13% of patients had scores ≥2 points, indicating severe symptoms) and 1.6 on the FFS scale (26% had scores ≥2 points, indicating poor function) [30].

In a study by Michelotti et al. conducted 2 years after surgery, the SSS score was 2.63 before surgery and 1.23 after surgery, whereas the FSS score was 2.24 and 1.16 before and after surgery, respectively [40].

## 5. Study Limitations

Notably, this study has some limitations which should be mentioned. The diagnosis of CTS was based on history and clinical symptoms. The purpose of this study was not to verify the diagnosis of CTS and analyze electroneurographic (ENG) or electromyographic (EMG) results, but to answer the question of how effective surgical treatment is in CCTS cases. We now also believe that it would be valuable to complete the BCTQ preoperatively as well.

We considered the functional hand grip test to be more reliable than manual muscle testing of the abductor pollicis brevis muscle. The patients in our study represented different degrees of thenar muscle atrophy; therefore, we decided on a simple evaluation of this symptom in a two-stage (0—present or 1—absent) score. Moreover, we used global grip strength evaluation, which is more common in the literature, but it is questioned as inferior to pinch grip measurements.

Another issue is the deliberate omission of an analysis of the conservative treatment applied. Such information on whether all patients were treated with physical methods, steroid injections, or wrist orthoses could have been an additional value of the paper, which would be especially of interest to physiotherapists. However, assuming that conservative methods were ineffective, these data were not analyzed in this study.

## 6. Conclusions

Patients with chronic CTS report better hand function after surgery despite the long duration of symptoms. Selected parameters (thenar muscles degeneration, global hand grip strength, and characteristic symptoms such as numbness or pain) were improved or retreated, but sensory resolution after surgery did not change and was similar to pre-surgery values.

The severity of symptoms and hand function after surgery improved relative to the preoperative results, and the results of questionnaires assessing this function were satisfactory.

The surgical treatment of patients with chronic carpal tunnel syndrome provided results according to which the majority of respondents were satisfied, and the findings from the self-report questionnaire indicated a reduction in the discomfort experienced before surgery.

## Figures and Tables

**Table 1:** The mean age of the patients studied, the duration of the disease (expressed in years), and the time from surgery to follow-up (expressed in months).

	Mean	Minimum	Maximum	SD
Age (N = 86)	57.76	34	85	8.71
Disease duration	8.47	5	20	3.08
Follow-up	33.48	12	65	7.009

Abbreviations: N, group size; SD, standard deviation.

**Table 2:** Results of the grip strength analysis, expressed in kilograms.

Variable	Group	N	Median	Q_1_	Q_3_	*p*
Grip strength before surgery		80	15.58	10.50	20.50	0.000001
Grip strength after surgery		86	22.35	16.37	25.83	
Grip strength before surgery	w	63	14.50	10.20	19.00	0.000001
Grip strength after surgery	w	69	21.20	15.20	24.57	
Grip strength before surgery	m	17	20.70	16.20	34.83	0.102435
Grip strength after surgery	m	17	27.53	23.97	34.27	
Grip strength before surgery	<60 years	45	15.50	10.27	22.07	0.000297
Grip strength after surgery	<60 years	48	24.37	16.97	27.52	
Grip strength before surgery	>60 years	35	15.67	10.67	20.10	0.000786
Grip strength after surgery	>60 years	38	20.95	15.83	23.87	

Abbreviations: N, group size; Q_1_, lower quartile; Q_3_, upper quartile; w, women; m, men.

**Table 3:** Results of the analysis of the discrimination of sensation on fingers I–III expressed in millimeters.

Variable	Group	N	Median	Q_1_	Q_3_	*p*
2PD before surgery		84	5.38	4.50	7.63	0.073486
2PD after surgery		85	5.00	4.50	6.00	
2PD before surgery	w	67	6.00	4.50	8.00	0.026250
2PD after surgery	w	68	5.00	4.38	6.00	
2PD before surgery	m	17	5.25	4.50	6.50	0.334278
2PD after surgery	m	17	6.00	5.25	6.50	
2PD before surgery	<60 years	47	5.25	4.50	7.50	0.009552
2PD after surgery	<60 years	47	5.00	4.00	5.25	
2PD before surgery	>60 years	37	6.25	4.50	8.00	0.825912
2PD after surgery	>60 years	38	6.00	5.00	6.75	

Abbreviations: N, group size; Q_1_, lower quartile; Q_3_, upper quartile; w, women; m, men.

**Table 4:** Results of the DASH questionnaire analysis.

Variable	Group	N	Median	Q_1_	Q_3_	*p*
Score before surgery		86	47.92	35.00	57.50	0.000000
Score after surgery		86	8.33	0.00	23.33	
Score before surgery	w	69	45.00	35.83	57.50	0.000000
Score after surgery	w	69	11.67	0.00	25.00	
Score before surgery	m	17	50.83	35.00	55.83	0.000293
Score after surgery	m	17	2.50	0.00	8.33	
Score before surgery	<60 years	48	46.25	35.00	60.83	0.000000
Score after surgery	<60 years	48	2.50	0.00	21.25	
Score before surgery	>60 years	38	49.58	35.83	56.67	0.000000
Score after surgery	>60 years	38	13.33	0.83	23.33	

Abbreviations: N, group size; Q_1_, lower quartile; Q_3_, upper quartile; w, women; m, men.

**Table 5:** Results of the analysis of the PRWE questionnaire.

Variable	Group	N	Median	Q_1_	Q_3_	*p*
PRWE before surgery		86	55.25	42.50	68.00	0.000000
PRWE after surgery		86	4.50	0.00	23.50	
PRWE before surgery	w	69	57.00	43.50	68.00	0.000000
PRWE after surgery	w	69	5.00	0.00	27.00	
PRWE before surgery	m	17	44.50	33.00	66.00	0.000293
PRWE after surgery	m	17	1.00	0.00	10.00	
PRWE before surgery	<60 years	48	57.00	45.25	68.00	0.000000
PRWE after surgery	<60 years	48	2.50	0.00	12.75	
PRWE before surgery	>60 years	38	47.25	40.00	66.00	0.000006
PRWE after surgery	>60 years	38	9.50	1.00	27.00	

Abbreviations: N, group size; Q_1_, lower quartile; Q_3_, upper quartile; w, women; m, men.

**Table 6:** Results of the BCTQ analysis.

Scales	Group	N Valid	Median	Q_1_	Q_3_	*p*
SSS		77	1.09	1.00	1.64	0.000000
FSS		77	1.25	1.00	2.00	0.000001
SSS	w	66	1.18	1.00	1.64	0.000000
FSS	w	66	1.25	1.00	2.38	0.000005
SSS	m	11	1.00	1.00	1.45	0.008599
FSS	m	11	1.13	1.00	1.25	0.000352
SSS	<60 years	45	1.00	1.00	1.36	0.000000
FSS	<60 years	45	1.25	1.00	1.88	0.000000
SSS	>60 years	32	1.32	1.05	1.73	0.012840
FSS	>60 years	32	1.25	1.00	2.44	0.010862

Abbreviations: FSS, Functioning Status Scale; SSS, Symptom Severity Scale; w, women; m, men; N, group size; Q_1_, lower quartile; Q_3_, upper quartile. P-normality test for each group.

**Table 7:** Results of the correlation analysis.

Correlation	N	r	*p*
Duration of symptoms & DASH	86	−0.01	0.951
Duration of symptoms & PRWE	86	−0.04	0.740
Duration of symptoms & BCTQ	77	0.05	0.662
Follow-up & DASH	86	**−0.26**	**0.014**
Follow-up & PRWE	86	**−0.29**	**0.006**
Follow-up & BCTQ	77	**−0.24**	**0.004**

Abbreviations: N, number of patients; r, Spearman’s rank correlation coefficient; *p*, *p* value; Follow-up, number of months between surgery and follow-up. Bold letters indicate significant correlations.

## Data Availability

Data available on request due to restrictions e.g., privacy or ethical.
